# Altered miRNA profile in testis of post-cryptorchidopexy patients with non-obstructive azoospermia

**DOI:** 10.1186/s12958-018-0393-3

**Published:** 2018-08-13

**Authors:** Dongdong Tang, Zhenyu Huang, Xiaojin He, Huan Wu, Dangwei Peng, Li Zhang, Xiansheng Zhang

**Affiliations:** 1 Reproductive Medicine Center, Department of Obstetrics and Gynecology, The First Affiliated Hospital of Anhui Medical University, Hefei, Anhui People’s Republic of China; 2 Anhui Province Key Laboratory of Reproductive Health and Genetics, Anhui Medical University, Hefei, Anhui People’s Republic of China; 3 Anhui Provincial Engineering Technology Research Center for Biopreservation and Artificial Organs, Hefei, Anhui People’s Republic of China; 4 Department of Urology, The First Affiliated Hospital of Anhui Medical University, Hefei, Anhui People’s Republic of China

**Keywords:** miRNA, Cryptorchidism, Cryptorchidopexy, Spermatogenesis, Next-generation small RNA sequencing

## Abstract

**Background:**

Cryptorchidism is one of the most common causes of non-obstructive azoospermia (NOA) leading to male infertility. Despite various medical approaches been utilised, many patients still suffer from infertility. MicroRNAs (miRNAs) play vital roles in the progress of spermatogenesis; however, little is known about the miRNA expression profile in the testes. Therefore, the miRNA profile was assessed in the testis of post-cryptorchidopexy patients.

**Methods:**

Three post-cryptorchidopexy testicular tissue samples from patients aged 23, 26 and 28 years old and three testis tissues from patients with obstructive azoospermia (controls) aged 24, 25 and 36 years old were used in this study. Next-generation sequencing (NGS) was used to perform the miRNA expression profiling. Quantitative real-time reverse transcription polymerase chain reaction (qRT-PCR) assays were subsequently used to confirm the results of several randomly-selected and annotated miRNAs.

**Results:**

A series of miRNAs were found to be altered between post-cryptorchidopexy testicular tissues and control tissues, including 297 downregulated and 152 upregulated miRNAs. In the subsequent qRT-PCR assays, the expression levels of most of the selected miRNAs (9/12, *P* < 0.05) were consistent with the results of NGS technology. Furthermore, signal transduction, adaptive immune response and biological regulation were associated with the putative target genes of the differentially-expressed miRNAs via GO analysis. In addition, oxidative phosphorylation, Parkinson’s disease and ribosomal pathways were shown to be enriched using KEGG pathway analysis of the differentially-expressed genes.

**Conclusions:**

This study provides a global view of the miRNAs involved in post-cryptorchidopexy testicular tissues as well as the altered expression of miRNAs compared to control tissues, thus confirming the vital role of miRNAs in cryptorchidism.

**Electronic supplementary material:**

The online version of this article (10.1186/s12958-018-0393-3) contains supplementary material, which is available to authorized users.

## Background

Male factors account for approximately 50% of infertility cases, which affect 10–15% of couples around the world [1]. Although most cases of male infertility are idiopathic with no known etiological factor, some causes (i.e. varicocele, sexual dysfunction etc.) are known [2]. Among these causes, cryptorchidism is a relatively common anomaly in the male genitalia that affects approximately 2–4% of male infants. Despite various medical approaches (i.e. surgical operations and hormone administration) being applied for years, many patients still suffer from infertility [3, 4], and little is known about the clear mechanism of spermatogenesis arrest in these patients.

Spermatogenesis is a complex process consisted of three phases including mitotic, meiotic and haploid processes [5]. These cellular events require highly regulated spatiotemporal expression of specific protein-coding genes, especailly at the post-transcriptional levels [6]. MicroRNAs (miRNAs) are a series of small noncoding RNAs that negatively regulate gene expression after transcription [7]. Research has shown that miRNAs play crucial roles in spermatogenesis [5, 6, 8–13]; for example, Lian et al. identified a series of altered miRNAs in patients with non-obstructive azoospermia (NOA) using microarray technology. These identified 154 significantly downregulated and 19 upregulated miRNAs indicated the important role of miRNAs in spermatogenesis [10]. It was reported that during mouse testicular development, up-regulation of miR-449 coincided with initiation of meiotic, and miR-449 was predominantly expressed in spermatocytes and spermatids during adult spermatogenesis. Furthermore, Cdc20b/miR-449 cluster activity was documented to be cooperatively mediated by CREMT and SOX5 during postnatal testes development [5]. Later on, Comazzetto et al. have identified the miR-34 family consisted of miR-34b/c and miR-449a/b/c as upregulated from late meiosis to sperm stage. miR-34b/c and miR-449 deletion led to sterility due to abnormal spermatozoa production with reduced motility [11]. With regards to the effects of miRNAs in cryptorchidism, Duan et al. found that miR-210, a significantly upregulated miRNA in patients with NOA, was also highly expressed in patients with cryptorchidism [12]. In addition, Moritoki et al. demonstrated that miR-135a was downregulated in unilateral undescended testes in a rat model of cryptorchidism [13].

Although some miRNAs were shown to be involved in the regulation of spermatogenesis in patients with cryptorchidism, no studies have yet investigated miRNA expression in the testis of post-cryptorchidopexy patients with NOA. Therefore this study investigated the miRNA profile in the testis of post-cryptorchidopexy patients and aimed to provide a platform to expound the mechanism of spermatogenesis arrest in post-cryptorchidopexy patients with NOA.

## Methods

### Ethics statement

Three patients (23, 26 and 28 years old) who underwent cryptorchidopexy but were still experiencing NOA, as well as three patients (24, 25 and 36 years old) suffering from obstructive azoospermia (OA) signed informed consent and approved the use of their tissues for research purposes. The local medical ethics committee approved this study.

### Clinical specimen collection

Testes tissues were collected by testicular biopsy from all six subjects between July 2017 and January 2018 at the Reproductive Medicine Center, First Affiliated Hospital of Anhui Medical University (Hefei, Anhui, China). For post-cryptorchidopexy patients, all cases were bilateral. Case one was 23 years old and underwent the operation 1 year ago, case two was 26 years old and underwent the operation 18 years ago and case three was 28 years old and underwent the operation 12 years ago. Testes samples were frozen at − 80 °C in RNAlater (Ambion, USA) immediately after surgery. Haematoxylin and eosin (HE) staining and the Johnson score system were used to assess testicular spermatogenic function.

### Construction of a smRNA library and next-generation sequencing (NGS)

Total RNA was extracted from the six samples using TRIzol (Life Technologies, USA) and was used to construct miRNA libraries using the NEBNext® Multiplex Small RNA Library Prep Set (Illumina®) according to the manufacturer’s instructions. Sequencing was performed on a Hiseq X (Illumina) using the HiSeq X Reagent Kit v2.

### Data analyses and novel miRNA exploration

Data were analysed according to previously-reported methods. Known miRNAs were identified by mapping reads to miRBase (version 21.0) in *Homo sapiens*, whilst nonmatched reads were subsequently aligned against other noncoding RNAs within the Ensembl database [14]. The remaining nonannotated sequences were selected for alignment with the integrated human transcriptome to explore novel miRNAs. All hairpin-like structures containing unclassified smRNA reads (no less than 45 reads) were predicted using miRDeep2 [15] following the criteria described previously [16].

### Bioinformatic analyses for miRNAs with differential expression patterns

The target genes of the differentially-expressed miRNAs in the two groups were predicted using TargetScan [17] and miRanda [18]. Enriched GO terms and KEGG pathway analysis was subsequently applied to predict the target genes of miRNAs with differential expression patterns in the two groups of specimens.

### QRT-PCR verification for altered miRNA expression

cDNA synthesis was performed using a PrimeScript RT reagent kit following the manufacturer’s instructions (Takara, Japan). The abundance of individual miRNAs was subsequently assessed via an Applied Biosystems 7500 PCR System (Applied Biosystems) using SYBR Premix Ex Taq II (Tli RNaseH Plus, Takara) under optimised reaction conditions. The specific reverse transcription and qPCR primers for all miRNAs are listed in Additional file 1. The processes were performed in accordance with the protocols supplied by the manufacturers. Briefly, for qPCR, triplicate reactions were performed at 95 °C for 10 min, and the subsequent 40 amplification cycles were conducted at 95 °C for 15 s and 60 °C for 60 s. Meanwhile, 18S rRNA was used as an internal normalised control. Relative miRNA abundances were calculated using 2^−△△Ct^ (threshold cycle) formula, where △Ct = Ct_miRNA_ − Ct_18S rRNA_ and △△Ct = (△Ct_post-cryptorchidopexy_ − △Ct_obstructive azoospermia_). The miRNA concentration differences between post-cryptorchidopexy and control tissues were analysed using unpaired t-tests. *P* < 0.05 indicated a statistically significant difference.

## Results

### Histopathological characteristics of post-cryptorchidopexy testicular tissue and control tissue

**Fig. 1:**
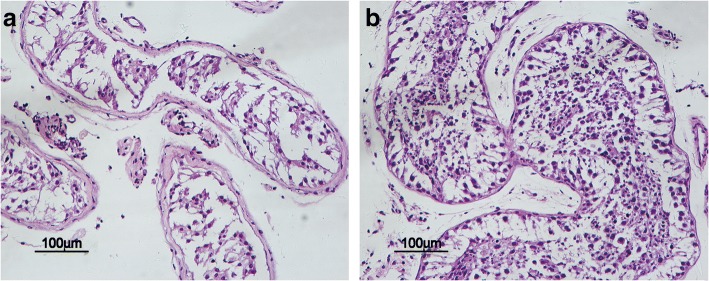
HE-staining of cryptorchid tissue and control tissue, which clarify the histopathological characteristics of cryptorchid tissues (**a**) and control tissues (**b**).

To clarify the histopathological characteristics of the post-cryptorchidopexy testicular tissue (hereafter referred to as ‘cryptorchid tissue’) and control tissue (hereafter referred to as ‘normal tissue’), HE staining and the Johnson scoring system were used to assess the function of spermatogenesis (Fig. 1). The Johnson scores were 3, 3 and 3 in cryptorchid tissues, which indicated maturation arrest, and 9, 9 and 10 in normal control tissues, which indicated normal spermatogenesis.

### Comprehensive overview of whole genome smRNAs in cryptorchid and normal tissues

**Fig. 2:**
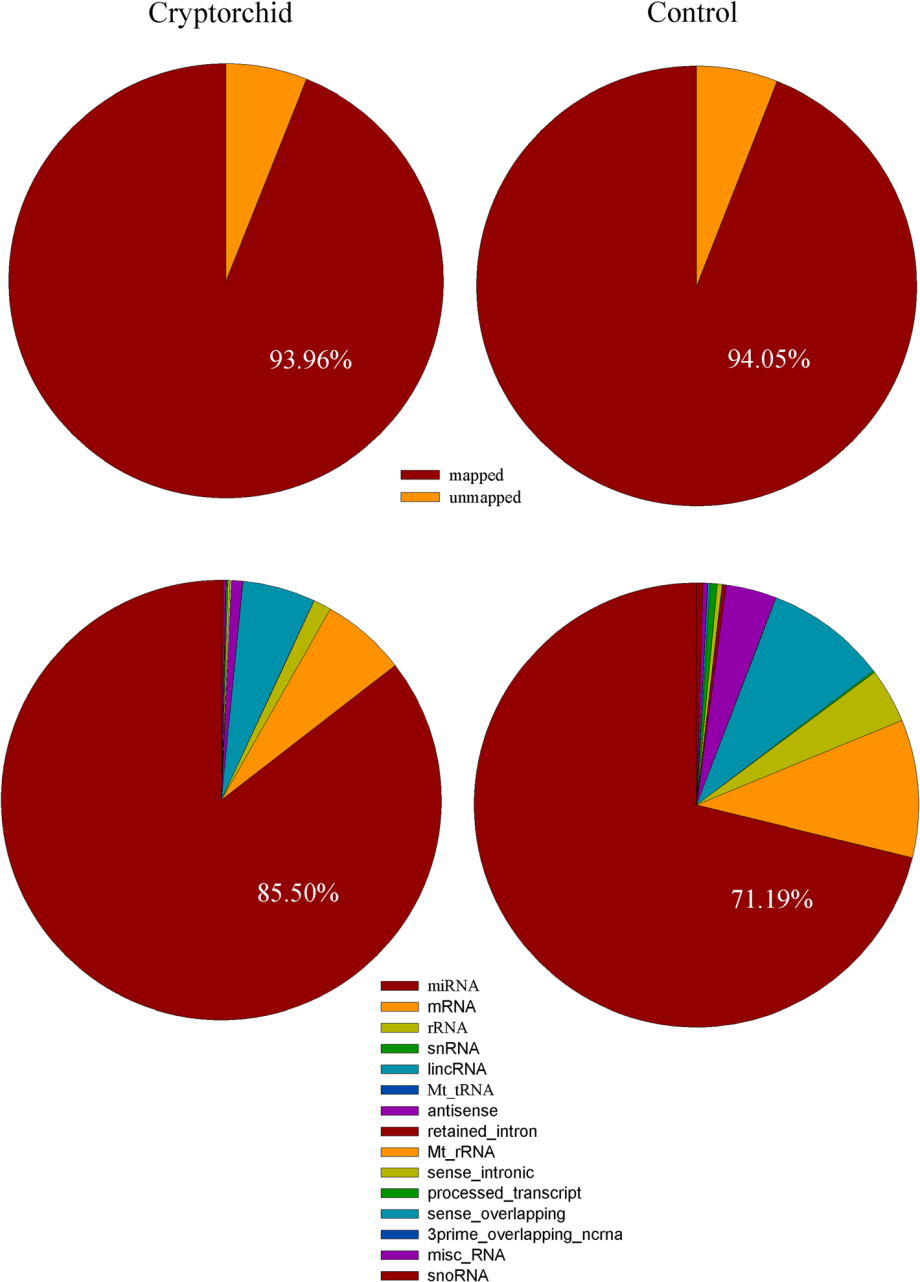
Results of geneome mapping and distribution of RNAs among different categories.

All smRNAs [18–32 nucleotides (nt)] acquired from cryptorchid and normal tissues were deep sequenced by NGS. A total of 19,931,698 (out of 21,212,215) and 20,243,124 (out of 21,524,351) sequence reads that aligned to the human genome sequence dataset were obtained in the cryptorchid and normal tissues, respectively. MiRNAs accounted for 85.5% and 71.19% in cryptorchid and normal tissues, respectively (Fig. 2).

The most abundant of these smRNAs in cryptorchid tissue were 21 nt in length, and these smRNAs were more abundant than the 22-nt and 23-nt RNAs which were in second and third place, respectively. However, the most abundant smRNAs in normal tissue were 22 nt in length, and these were more abundant than the 21-nt and 23-nt RNAs which were in second and third place, respectively (see Additional file 2).

Understanding the distribution pattern of miRNA genes may help to elucidate their roles, therefore the chromosomal locations of miRNA genes were evaluated. In cryptorchid tissue, most miRNA genes were located on chromosomes X, 9, 3 and 21. Similarly, in normal tissues, most miRNA genes were located on chromosome X, 15, 9 and 5 (see Additional file 3).

### Features of the most abundant miRNAs in cryptorchid and normal tissues

The NGS results were used to compile a list of the 20 most abundant and known miRNAs in cryptorchid tissue and the 10 most abundant and novel miRNAs in normal tissue. In cryptorchid tissue, miR-514a-3p, miR-143-3p, miR-26a-5p, miR-99a-5p, miR-202-5p, miR-509-3-5p, miR-10b-5p, miR-508-3p, let-7 g-5p and let-7f-5p were the most abundant known miRNAs. In normal tissue, miR-514a-3p, miR-143-3p, miR-26a-5p, miR-509-3-5p, miR-99a-5p, miR-202-5p, miR-10b-5p, let-7f-5p, miR-508-3p and let-7 g-5p were the most abundant known miRNAs (Table 1). Detailed information is shown in Table 1. Of the 10 most abundant novel miRNAs, only one was different between cryptorchid and normal tissues. Detailed information is shown in Table 2.

**Table 1:** The top 20 most abundant known miRNAs expressed in cryptorchid and normal tissues

miRNA name	Cryptorchid		miRNA name	Control	
	Reads count	Normalized reads count		Reads count	Normalized reads count
hsa-miR-514a-3p	2,313,282	109,499	hsa-miR-514a-3p	1,008,914	98,435
hsa-miR-143-3p	864,140	45,306	hsa-miR-143-3p	677,433	65,245
hsa-miR-26a-5p	829,953	46,035	hsa-miR-26a-5p	407,763	39,613
hsa-miR-99a-5p	705,575	38,041	hsa-miR-509-3-5p	392,074	37,711
hsa-miR-202-5p	616,580	30,770	hsa-miR-99a-5p	346,310	33,871
hsa-miR-509-3-5p	593,363	28,054	hsa-miR-202-5p	258,855	25,790
hsa-miR-10b-5p	428,806	24,984	hsa-miR-10b-5p	248,352	24,218
hsa-miR-508-3p	303,445	14,625	hsa-let-7f-5p	154,806	15,116
hsa-let-7 g-5p	296,741	15,472	hsa-miR-508-3p	153,631	15,059
hsa-let-7f-5p	266,118	14,707	hsa-let-7 g-5p	151,745	14,851
hsa-let-7a-5p	265,013	14,644	hsa-let-7a-5p	137,886	13,412
hsa-miR-21-5p	248,959	12,378	hsa-miR-21-5p	132,197	12,931
hsa-miR-509-5p	194,212	9293	hsa-miR-148a-3p	119,576	11,671
hsa-miR-148a-3p	188,828	10,645	hsa-miR-100-5p	103,191	10,071
hsa-miR-125b-5p	172,432	9013	hsa-miR-125b-5p	93,627	9134
hsa-miR-100-5p	169,375	9160	hsa-miR-27b-3p	92,637	8927
hsa-miR-199a-3p	154,971	8592	hsa-miR-509-5p	81,898	8124
hsa-miR-27b-3p	144,132	7610	hsa-miR-126-3p	79,980	7687
hsa-let-7i-5p	140,689	7772	hsa-miR-125a-5p	72,130	7013
hsa-let-7b-5p	112,327	6040	hsa-miR-34c-5p	69,568	6885
hsa-miR-125a-5p	107,449	5594	hsa-let-7i-5p	66,915	6560

**Table 2:** The list of top 10 most abundant novel miRNAs expressed in cryptorchid and normal tissues

Cryptorchid tissues			
miRNA ID	Mature Sequence	Reads count	Location of novel miRNA precusor
chrX_47246	AUUGACACUUCUGUGAGUAGA	2,280,438	chrX:146366172..146366230:-
chr12_27425	UUCAAGUAAUCCAGGAUAGGCU	826,714	chr12:58218403..58218462:-
chr3_5958	UUCAAGUAAUCCAGGAUAGGCU	826,558	chr3:38010903..38010964:+
chr21_44054	AACCCGUAGAUCCGAUCUUGU	693,017	chr21:17911420..17911480:+
chrX_47235	UACUGCAGACGUGGCAAUCAUG	592,879	chrX:146341178..146341235:-
chr10_23103	UUCCUAUGCAUAUACUUCUUU	586,995	chr10:135061041..135061097:-
chr5_9937	UGAGAUGAAGCACUGUAGCUC	534,462	chr5:148808506..148808561:+
chr2_3766	UACCCUGUAGAACCGAAUUUGU	428,617	chr2:177015056..177015117:+
chrX_47228	UGAUUGUAGCCUUUUGGAGUAGA	298,225	chrX:146318462..146318520:-
chr3_7283	UGAGGUAGUAGUUUGUACAGUU	295,643	chr3:52302295..52302373:-
Normal tissues			
miRNA ID	Mature Sequence	Reads count	Location of novel miRNA precusor
chrX_47246	AUUGACACUUCUGUGAGUAGA	996,346	chrX:146366172..146366230:-
chr5_9937	UGAGAUGAAGCACUGUAGCUC	419,103	chr5:148808506..148808561:+
chr12_27425	UUCAAGUAAUCCAGGAUAGGCU	405,817	chr12:58218403..58218462:-
chr3_5958	UUCAAGUAAUCCAGGAUAGGCU	405,621	chr3:38010903..38010964:+
chrX_47235	UACUGCAGACGUGGCAAUCAUG	391,755	chrX:146341178..146341235:-
chr21_44054	AACCCGUAGAUCCGAUCUUGU	340,009	chr21:17911420..17911480:+
chr2_3766	UACCCUGUAGAACCGAAUUUGU	248,236	chr2:177015056..177015117:+
chr10_23103	UUCCUAUGCAUAUACUUCUUU	246,558	chr10:135061041..135061097:-
chr9_18744	UGAGGUAGUAGAUUGUAUAGUU	154,925	chr9:96938634..96938712:+
chr3_7283	UGAGGUAGUAGUUUGUACAGUU	151,154	chr3:52302295..52302373:-

### Differential expression of miRNAs between cryptorchid and normal tissues

**Fig. 3:**
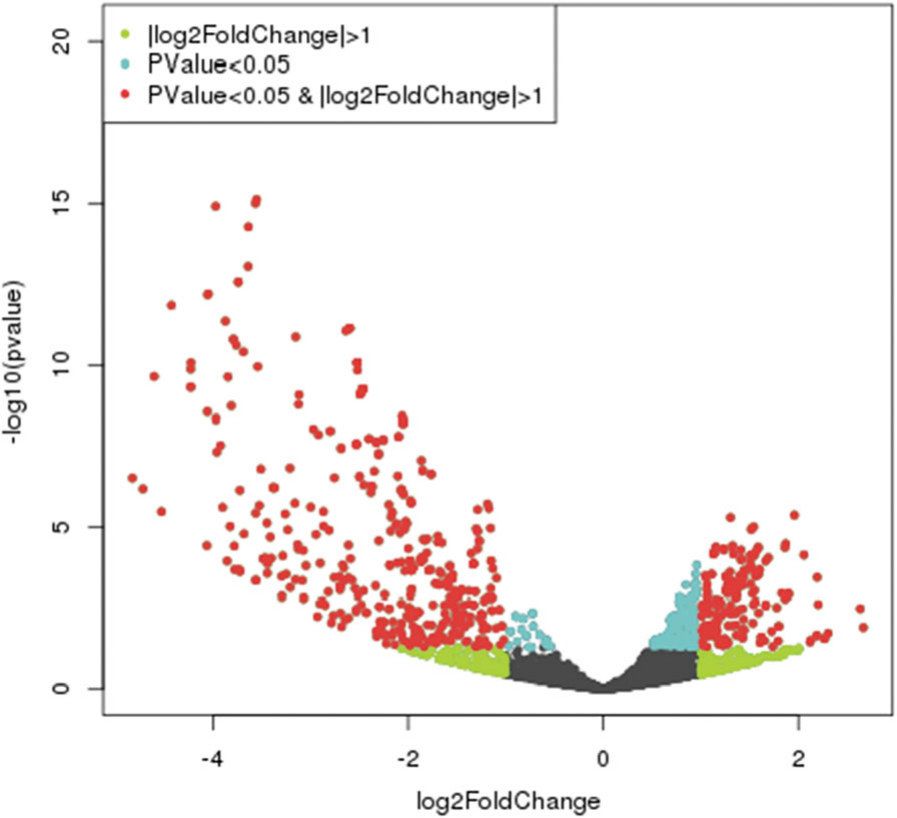
The overview of the volcano plot generated by miRNAs profile in cryptorchid tissues and control tissues.

As described previously by Zhang et al. [16], miRNAs were considered to be significantly differentially expressed between cryptorchid and normal tissues if they were altered by at least two-fold with *P <* 0.05 on the t-test. The results showed that 449 miRNAs were significantly differentially expressed in cryptorchid tissue (Fig. 3). Of these, 297 were downregulated and 152 were upregulated compared to normal tissue. The 30 most downregulated and upregulated known miRNAs are listed in Tables 3 and 4, respectively.

**Table 3:** A collection of the top 30 most downregulated known miRNAs detected by deep sequencing in cryptorchid tissues

MiRNA name	baseMean	log2FoldChange	lfcSE	stat	p	Adjust p
hsa-miR-3663-5p	41.936	−4.426	0.624	−7.089	1.35E-12	2.39E-10
hsa-miR-1233-3p	25.216	−4.227	0.679	−6.225	4.79E-10	1.84E-08
hsa-miR-552-5p	66.556	−4.055	0.563	−7.195	6.24E-13	1.21E-10
hsa-miR-449b-5p	392.523	−3.972	0.496	−8.001	1.23E-15	5.26E-13
hsa-miR-7153-5p	108.897	− 3.812	0.634	−6.010	1.84E-09	5.18E-08
hsa-miR-122-5p	525.785	−3.790	0.562	−6.741	1.57E-11	1.60E-09
hsa-miR-552-3p	65.189	−3.760	0.562	−6.680	2.38E-11	2.31E-09
hsa-miR-449a	5575.001	−3.740	0.511	−7.317	2.52E-13	5.97E-11
hsa-miR-122-3p	4.738	−3.722	1.011	−3.679	0.00023	0.0016
hsa-miR-34b-5p	123.524	−3.688	0.558	−6.610	3.84E-11	3.56E-09
hsa-miR-449c-5p	2234.173	−3.637	0.465	−7.816	5.42E-15	1.93E-12
hsa-miR-34c-5p	39,328.272	−3.553	0.440	−8.060	7.58E-16	5.26E-13
hsa-miR-449c-3p	7.961	−3.441	0.902	−3.812	0.00014	0.0011
hsa-miR-375	491.449	−3.408	0.362	−9.416	4.68E-21	9.99E-18
hsa-miR-3663-3p	37.612	−3.385	0.676	−5.001	5.68E-07	9.63E-06
hsa-miR-7159-5p	20.897	−3.259	0.705	−4.618	3.87E-06	5.29E-05
hsa-miR-449b-3p	142.460	−3.212	0.610	−5.262	1.42E-07	2.75E-06
hsa-miR-4700-5p	4.985	−3.208	0.951	−3.370	0.00075	0.0043
hsa-miR-522-3p	121.036	−3.153	0.465	−6.768	1.30E-11	1.46E-09
hsa-miR-1273a	38.566	−3.118	0.508	−6.135	8.47E-10	2.44E-08
hsa-miR-1295a	11.735	−3.075	0.760	−4.041	5.31E-05	0.0005
hsa-miR-34b-3p	1137.731	−2.970	0.516	−5.753	8.72E-09	2.16E-07
hsa-miR-1283	139.436	−2.798	0.488	−5.731	9.95E-09	2.41E-07
hsa-miR-3150b-3p	3.547	−2.768	0.991	−2.791	0.0052	0.020
hsa-miR-4423-3p	16.582	−2.702	0.755	−3.578	0.00035	0.0023
hsa-miR-6507-5p	7.696	−2.698	0.811	−3.325	0.00088	0.0049
hsa-miR-7154-5p	406.827	−2.646	0.981	−2.697	0.0070	0.025
hsa-miR-517c-3p	95.074	−2.639	0.386	−6.832	8.37E-12	9.92E-10
hsa-miR-3925-3p	10.324	−2.613	0.735	−3.553	0.00038	0.0025
hsa-miR-515-5p	84.007	−2.600	0.379	−6.856	7.04E-12	8.84E-10

**Table 4:** A collection of the top 30 most upregulated known miRNAs detected by deep sequencing in cryptorchid tissues

MiRNA name	baseMean	log2FoldChange	lfcSE	stat	p	Adjust p
hsa-miR-7151-3p	6.026	2.634	0.892	2.953	0.0031	0.014
hsa-miR-376a-2-5p	10.918	2.202	0.724	3.042	0.0023	0.011
hsa-miR-1224-5p	17.708	2.193	0.615	3.565	0.00036	0.0024
hsa-miR-1299	187.854	1.958	0.426	4.600	4.22E-06	5.73E-05
hsa-miR-142-5p	697.547	1.898	0.583	3.255	0.0011	0.0060
hsa-miR-543	1281.559	1.869	0.450	4.152	3.29E-05	0.00036
hsa-miR-487a-3p	80.564	1.865	0.591	3.155	0.0016	0.0079
hsa-miR-584-3p	19.666	1.829	0.562	3.254	0.0011	0.0060
hsa-miR-665	18.416	1.798	0.710	2.534	0.011	0.036
hsa-miR-134-3p	29.541	1.778	0.598	2.975	0.0029	0.013
hsa-miR-369-3p	500.851	1.692	0.432	3.916	8.99E-05	0.00082
hsa-miR-377-3p	96.245	1.665	0.551	3.023	0.0025	0.011
hsa-miR-33a-5p	28.103	1.664	0.550	3.025	0.0025	0.011
hsa-miR-376a-3p	112.0733	1.602	0.436	3.704	0.00021	0.0015
hsa-miR-758-3p	520.1303	1.589	0.439	3.620	0.00029	0.0020
hsa-miR-654-3p	4175.568	1.587	0.388	4.095	4.22E-05	0.00044
hsa-miR-134-5p	2747.859	1.558	0.424	3.675	0.00024	0.0017
hsa-miR-889-3p	740.3619	1.552	0.468	3.312	0.00093	0.0052
hsa-miR-127-3p	40,871.646	1.548	0.392	3.955	7.65E-05	0.00071
hsa-miR-1185-1-3p	161.457	1.539	0.506	3.039	0.0024	0.011
hsa-miR-1185-2-3p	38.541	1.534	0.587	2.614	0.0089	0.030
hsa-miR-154-5p	267.267	1.516	0.346	4.385	1.16E-05	0.00014
hsa-miR-381-3p	7512.422	1.511	0.382	3.957	7.57E-05	0.00070
hsa-miR-127-5p	768.176	1.511	0.401	3.765	0.00017	0.0013
hsa-miR-337-5p	44.570	1.510	0.439	3.437	0.00059	0.0036
hsa-miR-379-3p	262.022	1.508	0.401	3.756	0.00017	0.0013
hsa-miR-136-3p	937.135	1.506	0.389	3.868	0.00011	0.00096
hsa-miR-376c-3p	327.216	1.492	0.402	3.713	0.00020	0.0015
hsa-miR-495-3p	884.797	1.443	0.390	3.696	0.00022	0.0016
hsa-miR-376b-5p	24.828	1.442	0.590	2.445	0.014	0.045

### Validating the altered expression level of miRNAs by qRT-PCR

**Fig. 4:**
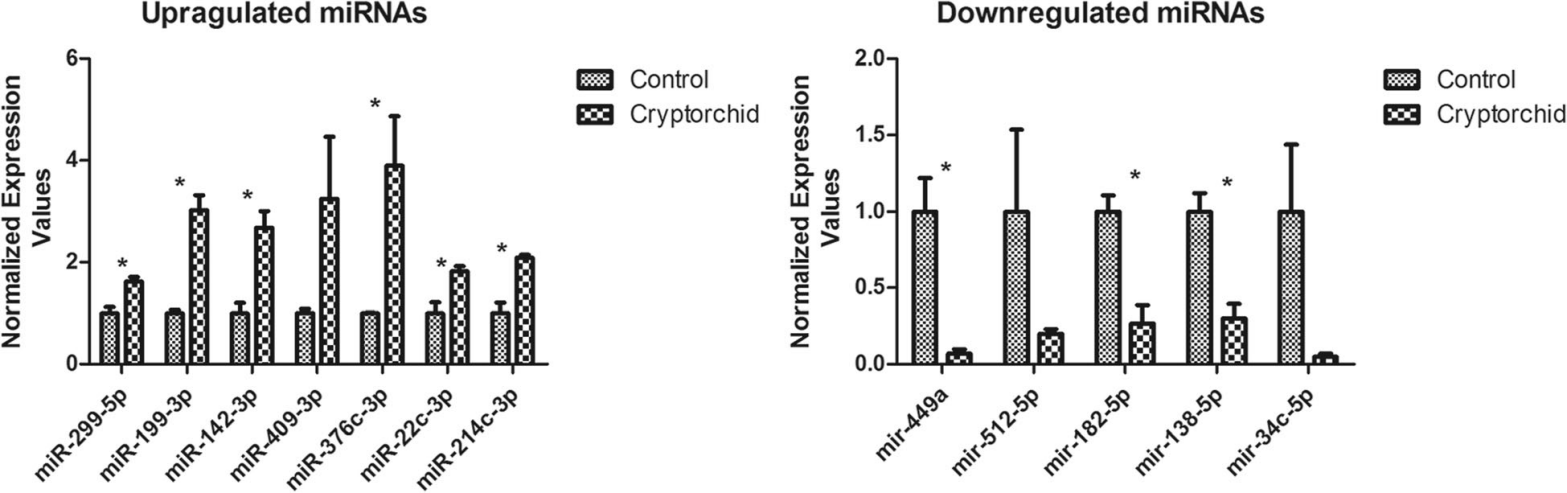
Confirmation of differentially expressed miRNAs between cryptorchid tissues and control tissues obtained by NGS using qRT-PCR. (* *P* <0.05)

QRT-PCR was performed to validate the altered miRNA expression. Among these deregulated miRNAs, we firstly selected two well-established spermatogenesis-associated miRNAs, miR-449a and miR-34c-5p [5, 11]. Additionally, to better proving the accuracy of NGS, the other validated miRNAs were picked from the non-top 30 most deregulated known miRNAs (see Additional file 4 and Additional file 5), so that the relatively small fold changes could be validated. According to the previous studies, ten miRNAs were picked for qRT-PCR validation randomly [16, 19]. Eventually, a total of 12 differentially-expressed miRNAs (seven upregulated and five downregulated) were selected for qRT-PCR analysis. The results showed that the expression levels of most miRNAs (9/12; *P* < 0.05) were consistent with the results of NGS technology. Detailed information is shown in Fig. 4.

### GO enrichment analysis of differentially-expressed genes in cryptorchid and normal tissues

After predicting the target genes of differentially-expressed miRNAs in cryptorchid and normal tissues, GO enrichment analysis was conducted. The 10 most enriched GO terms, including signal transduction and adaptive immune response, are shown in Table 5.

**Table 5:** Top 30 most enriched GO terms for predicted targets of differentially expressed miRNAs between cryptorchid and normal tissues

GO number	Term^*^	GO process	Ratio in study	Ratio in pop	p
GO:0007165	BP	signal transduction	19.68%	23.63%	1.04E-05
GO:0002250	BP	adaptive immune response	0.70%	1.83%	1.56E-05
GO:0050789	BP	regulation of biological process	47.95%	52.34%	4.14E-05
GO:0050794	BP	regulation of cellular process	45.10%	49.34%	7.66E-05
GO:0008150	BP	biological_process	78.42%	81.73%	8.25E-05
GO:0065007	BP	biological regulation	51.05%	55.25%	8.51E-05
GO:0006956	BP	complement activation	0.25%	0.98%	0.000114
GO:0006958	BP	complement activation, classical pathway	0.20%	0.88%	0.000134
GO:0048518	BP	positive regulation of biological process	21.53%	25.03%	0.00014
GO:0050776	BP	regulation of immune response	3.95%	5.72%	0.000229
GO:0044425	CC	membrane part	28.37%	34.86%	1.03E-10
GO:0005886	CC	plasma membrane	17.68%	23.38%	1.11E-10
GO:0031224	CC	intrinsic component of membrane	23.23%	29.30%	2.06E-10
GO:0016021	CC	integral component of membrane	22.68%	28.69%	2.24E-10
GO:0005575	CC	cellular_component	84.22%	88.01%	1.38E-07
GO:0005794	CC	Golgi apparatus	3.35%	5.84%	1.42E-07
GO:0005840	CC	ribosome	2.20%	1.09%	7.38E-06
GO:0000139	CC	Golgi membrane	1.85%	3.41%	1.92E-05
GO:0044459	CC	plasma membrane part	9.84%	12.81%	1.93E-05
GO:0004872	MF	receptor activity	5.39%	8.48%	5.53E-08
GO:0060089	MF	molecular transducer activity	5.39%	8.48%	5.53E-08
GO:0005179	MF	hormone activity	1.55%	0.54%	6.49E-08
GO:0004871	MF	signal transducer activity	5.79%	8.78%	2.25E-07
GO:0038023	MF	signaling receptor activity	4.45%	7.13%	3.21E-07
GO:0099600	MF	transmembrane receptor activity	4.25%	6.85%	4.09E-07
GO:0003823	MF	antigen binding	0.50%	1.75%	4.18E-07
GO:0004888	MF	transmembrane signaling receptor activity	4.15%	6.63%	9.33E-07
GO:0032553	MF	ribonucleotide binding	6.34%	8.94%	1.10E-05
GO:0003674	MF	molecular_function	77.82%	81.17%	7.79E-05

*BP Biological process; *CC* Cellular component; *MF* Molecular function

### KEGG pathway analysis of differentially-expressed genes in cryptorchid and normal tissues

After GO analysis, KEGG pathway enrichment analysis was performed. A total of five KEGG pathways were enriched, including oxidative phosphorylation, Parkinson’s disease, Ribosomal pathways, Huntington’s disease and Alzheimer’s disease. The results are presented in Table 6.

**Table 6:** KEGG pathway analysis for predicted target genes of differentially expressed miRNAs between cryptorchid and normal tissues

Pathway ID	Description	GeneRatio	BgRatio	p	Adjust p	GeneName
hsa00190	Oxidative phosphorylation	28/664	133/7297	1.80E-05	0.0053	ATP5G2;COX6C;SDHD;COX7A2L; COX8C;ATP6V1D
hsa05012	Parkinson’s disease	28/664	142/7297	6.29E-05	0.0093	ATP5G2;COX6C;SDHD;UBB;UBE2L6;COX7A2L;GNAL;COX8C
hsa03010	Ribosome	29/664	154/7297	0.0001112	0.0110	MRPL16;RPL38;RPS4X;MRPL35; RPS6;MRPS18C;RPL26;RPS27L
hsa05016	Huntington’s disease	33/664	193/7297	0.0002633	0.0187	ATP5G2;COX6C;UCP1;SDHD; POLR2J3;COX7A2L;COX8C;POLR2K
hsa05010	Alzheimer’s disease	30/664	171/7297	0.0003147	0.0187	ATP5G2;COX6C;CASP12;SDHD; PPP3CC;COX7A2L;LPL;COX8C

## Discussion

As one of the most common congenital defects in newborn boys, cryptorchidism influences male fertility and increases the risk of testicular cancer. Reductions in seminiferous tubules and germ cells are common histological changes in cryptorchid testis [20]. Despite surgery being recommended for many patients with cryptorchidism, the success of orchidopexy depends on the timing of the procedure and the position of the testis: some may not benefit from cryptorchidopexy [21, 22]. Although research has identified some biological processes involved in spermatogenic arrest in cryptorchid testis (i.e. significant apoptotic changes in germ cells), the causative roles of genes in spermatogenic arrest or apoptosis remain unclear [23–26]. This is the first study to investigate the possible mechanisms of spermatogenic arrest in cryptorchid testes by assessing the miRNA profiles in post-cryptorchidopexy testes.

Many rodent and primate models were developed to identify altered miRNAs in cryptorchid testis. For example, Duan et al. established a mouse model of cryptorchidism and showed that miR-210 was highly expressed in cryptorchid testes compared with control testes. Moreover, they showed that this miRNA regulated spermatogenesis by inhibiting the expression of NR1D2 [12]. Moritoki et al. compared the miRNA expression profiles of unilateral undescended testes with contralateral descended testes in a rat model of cryptorchidism using microarray analysis. These authors found that only miR-135a expression was lower in unilateral undescended testes and that its target, FoxO1, played essential roles in stem cell maintenance [13]. Furthermore, Duan et al., also found that miR-210 was upregulated in human cryptorchidism, thus suggesting a vital role for miRNAs in humans [12]. In this study, 297 downregulated and 152 upregulated miRNAs were identified in post-cryptorchidopexy testicular tissue compared with normal testis tissue. However, miR-210 was not significantly altered, which may be due to the different types of human cryptorchid tissue. For example, Duan et al. used cryptorchid testis tissue obtained during the cryptorchidopexy, whilst this study used post-cryptorchidopexy testicular tissue. Some miRNA expression levels may change after the operation.

Despite the insights gained into cryptorchidism over the years, the mechanism of spermatogenesis arrest in patients with this disease remains largely elusive. Germ cell apoptosis is commonly seen at the histological level in cryptorchid testes. Yin et al. revealed that cryptorchidism induced germ cell apoptosis in an experimental mouse model via p53-dependent and p53-independent pathways [23]. Liu et al. also found that the Hsf1/Phlda1 pathway participated in primary spermatocyte apoptosis in surgery-induced cryptorchid testes of rats [27]. The expression of many apoptosis-related miRNAs was also shown to be altered in post-cryptorchidopexy testicular tissues. It was reported that miR-299-5p could modulate apoptosis through autophagy in neurons and ameliorate the cognitive capacity of APPswe/PS1dE9 mice [28]. In addition, miR-299-5p was significantly upregulated in post-cryptorchidopexy testicular tissue. Similar results were also found for miR-217, miR-206 etc. Li et al. also found that miR-217 could regulate apoptosis by targeting TNFSF11 in human podocyte cells [29]. This study also identified a significant downregulation of miR-217 in post-cryptorchidopexy testicular tissue. Similarly, miR-206 was significantly upregulated in post-cryptorchidopexy testicular tissue and was shown to promoted cell apoptosis in Legg–Calvé–Perthes disease [30].

## Conclusions

In summary, miRNA expression in post-cryptorchidopexy testicular tissue was profiled using NGS and compared with that of OA men with normal spermatogenesis. Several signalling pathways that are likely to be involved in spermatogenesis arrest in these patients were addressed. The results provide an important platform for future investigations into the roles of miRNAs in the progression of cryptorchidism as well as therapeutic targets to help these patients recover fertility. However, the comprehensive modulating behaviours of genes remain unclear, therefore determining the target genes and regulatory networks of these differentially-expressed miRNAs is essential in future investigations.

## Additional files

Additional file 1: Primers used for the quantification of representative deregulated miRNAs. (XLS 23 kb)

Additional file 2: Length distribution of clean reads from smRNA next-generation deep sequencing. (TIF 2836 kb)

Additional file 3: Number of smRNA sequencing tags that locate on each chromosome. (TIF 3575 kb)

Additional file 4: A collection of all the downregulated known miRNAs detected by deep sequencing in cryptorchid tissues. (XLS 45 kb)

Additional file 5: A collection of all the upregulated known miRNAs detected by deep sequencing in cryptorchid tissues. (XLS 35 kb)

## Abbreviations

HE: Hematoxylin-eosin
miRNAs: MicroRNAs
NGS: Next-generation sequencing
NOA: Non-obstructive azoospermia
OA: Obstructive azoospermia
qRT-PCR: Quantitative real-time reverse transcription-polymerase chain reaction
